# Relationship between FEV1 and Cardiovascular Risk Factors in General Population without Airflow Limitation

**DOI:** 10.1155/2016/8319849

**Published:** 2016-11-28

**Authors:** Jeong Hyeon Lee, Yun-Seong Kang, Yun-Jeong Jeong, Young-Soon Yoon, Won Gun Kwack, Jin Young Oh

**Affiliations:** ^1^Division of Pulmonary and Critical Care Medicine, Department of Internal Medicine, Dongguk University Ilsan Hospital, Goyang, Republic of Korea; ^2^Division of Pulmonary and Critical Care Medicine, Department of Internal Medicine, Seoul National University Hospital, Seoul, Republic of Korea

## Abstract

*Purpose.* We aimed to determine the value of lung function measurement for predicting cardiovascular (CV) disease by evaluating the association between FEV1 (%) and CV risk factors in general population.* Materials and Methods.* This was a cross-sectional, retrospective study of subjects above 18 years of age who underwent health examinations. The relationship between FEV1 (%) and presence of carotid plaque and thickened carotid IMT (≥0.8 mm) was analyzed by multiple logistic regression, and the relationship between FEV1 (%) and PWV (%), and serum uric acid was analyzed by multiple linear regression. Various factors were adjusted by using Model 1 and Model 2.* Results.* 1,003 subjects were enrolled in this study and 96.7% (*n* = 970) of the subjects were men. In both models, the odds ratio of the presence of carotid plaque and thickened carotid IMT had no consistent trend and statistical significance. In the analysis of the PWV (%) and uric acid, there was no significant relationship with FEV1 (%) in both models.* Conclusion.* FEV1 had no significant relationship with CV risk factors. The result suggests that FEV1 may have no association with CV risk factors or may be insensitive to detecting the association in general population without airflow limitation.

## 1. Introduction

Cardiovascular disease (CVD) is one of the main causes of mortality in the general population as well as in those with chronic obstructive pulmonary disease (COPD) [1]. Reduced lung function measured by forced vital capacity (FVC) or forced expiratory volume in one second (FEV1) is also related to increased risk of CVD in COPD [2–5].

Previous studies suggested that COPD and CVD might have this association because of their connection with regional or systemic inflammation [6–8]. Low-grade systemic inflammation was thought to be important in the pathogenesis and progression of atherosclerotic diseases [9] and actually systemic inflammatory markers such as hsCRP were significantly higher in COPD patients compared to healthy controls [10].

Currently, evaluation of CVD risk is commonly and easily done by measurement of carotid intima-media thickness (IMT) and pulse wave velocity (PWV). Increased carotid IMT or abnormal PWV is used as a predictor of subclinical atherosclerotic burden [11–14] and both have been associated with decreased lung function in COPD [10, 15]. Uric acid is also a CV risk factor and has recently been considered as a lung protective factor [16, 17].

Until now, most studies reporting the association of decreased lung function (FEV1, FVC) with CVD risk factors have been conducted in patients with moderate to severe COPD. Their association in the general population without airflow limitation has not been clearly defined.

This study aimed to determine the value of lung function measurement for predicting CVD by clarifying the relationship between decreased lung function represented by FEV1 and CV risk factors in general population without airflow limitation.

## 2. Materials and Methods

### 2.1. Study Population and Design

This was a cross-sectional, retrospective study of 1,260 subjects above 18 years of age who underwent health examinations at Dongguk University Ilsan Hospital (Goyang, Korea) between 01 March 2006 and 29 February 2012. The exams included a questionnaire of smoking status, pulmonary function test (PFT), carotid ultrasonography (US), PWV test, and determination of serum uric acid level. Exclusion criteria were as follows: no questionnaire (*n* = 16), indefinite smoking status (*n* = 47), FEV1 < 80% (*n* = 70), FEV1/FVC < 70% (*n* = 89), and ankle-brachial index (ABI) < 0.9 (*n* = 35). We divided the final enrolled subjects (*n* = 1,003) into four groups according to FEV1 (%). The study design was approved by the Institutional Review Board of the Dongguk University Ilsan Hospital.

### 2.2. Measurements

#### 2.2.1. Smoking Status and Body Mass Index

All participants filled self-administered questionnaires which included questions about smoking status. The patients' height and weight were obtained from the database and body mass index (BMI) was calculated as weight in kilograms divided by height squared (kg/m^2^).

#### 2.2.2. Blood Tests

Venous samples were collected after a 12 h overnight fast and serum white blood cell (WBC) count, serum lipids, high-sensitivity C-reactive protein (hsCRP), hemoglobin A1C (HbA1C), and serum uric acid were examined. The biochemical tests were done using a Roche/Hitachi cobas c702 automatic analyzer (Roche Diagnostics) at the Department of Laboratory Medicine at Dongguk University Ilsan Hospital, which is accredited by the Korean Society for Laboratory Medicine and the Korean Association of Quality Assurance.

#### 2.2.3. Spirometry

Spirometry was performed in the standing position by experienced technicians following the American Thoracic Society recommendations. A Vmax 2130 spirometer (Sensormedics) was used to measure FVC and FEV1 in absolute values (L), and predicted spirometry values (%) were calculated using the Morris equations [18]. Airflow limitation was defined as FEV1/FVC < 70% or FEV1 < 80% according to Global Initiative for Chronic Obstructive Lung Disease (GOLD) criteria [19]. Postbronchodilator spirometric test was not included in the health examination program. Subjects were classified in quartiles according to the FEV1 (% pred).

#### 2.2.4. Carotid Ultrasonography

Carotid ultrasonography was conducted by a registered sonographer by high-resolution ultrasound system (Vivid 7 or Vivid E9; GE). The carotid arteries were examined both sides and the longitudinal images were obtained at the bulb of common carotid artery (CCA) and the carotid IMT was measured in the straight segment of the CCA 20 mm proximal to the carotid bifurcation.

The thickened IMT was defined when both or one carotid IMT was ≥0.8 mm. The carotid IMT ≥ 0.8 mm was regarded as the cut-off of increased cardiovascular risk in previous studies [20, 21]. The carotid plaque was defined as carotid wall thickness > 1.2 mm [22, 23].

#### 2.2.5. PWV

Brachial-ankle PWV (baPWV) was obtained simply by wrapping blood pressure cuffs on the four extremities after at least a 5-minute rest and was calculated as the ratio of the virtual arterial path length derived from the subject's height and the time difference between the initiation point of systolic increases in brachial and ankle pressure waves. baPWV was measured using a Colin VP-2000 automated waveform analyzer (Omron Healthcare). The percentage of PWV (PWV%) represented the difference from the value of calculated pulse wave velocity which was based upon age-matched Asian population [23]. Subjects with an ABI <0.9 (*n* = 35) were excluded because baPWV can be underestimated when the subject has a peripheral arterial disease, which can be defined as ABI <0.9 [24, 25].

### 2.3. Statistical Analyses

We classified the subjects in quartiles by FEV1 (%): FEV1 80–93%, 94–100%, 101–109%, and 110–155%. To analyze subjects' baseline characteristics, we used one-way analysis of variance (ANOVA) for continuous variables and linear-by-linear association for categorical variables. The relationship between FEV1 and presence of carotid plaque or thickened IMT was analyzed by multiple logistic regression. The relationship between FEV1 and PWV (%) or serum uric acid was analyzed by simple and multiple linear regression. Model 1 was adjusted for age, BMI, and smoking status. Model 2 was adjusted for age, BMI, smoking status, low density lipoprotein (LDL) cholesterol, high density lipoprotein (HDL) cholesterol, systolic blood pressure (BP), HbA1C, and hsCRP. R version 3.2.4 (R foundation for statistical computing, Vienna, Austria) was used as the statistical software, for windows and results were considered as statistically significant when the *p* value was <0.05.

## 3. Results

### 3.1. Baseline Characteristics of the Study Population

**Figure 1:**
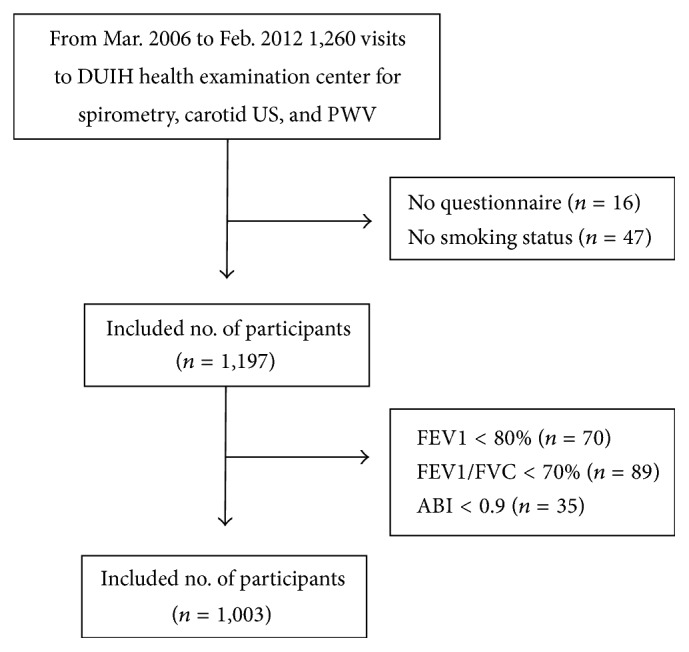
Baseline characteristics of the subjects.

Among the 1,260 participants who underwent health examinations that included spirometry, carotid US, PWV, and serum uric acid, 1,003 subjects were enrolled in this study (Figure 1). The baseline characteristics of the subjects are shown in Table 1. The mean age was 47.8 ± 9.27 years and 96.7% (*n* = 970) of the subjects were men. Among them, 72.9% (*n* = 731) were current or ex-smokers; the mean BMI was 24.46 ± 3.22 kg/m^2^ and mean uric acid was 6.04 ± 1.31 mg/dL.

We classified subjects in quartiles according to the FEV1 (%) (Table 2). Age increased as the FEV1 (%) increased. The age of the 4th quartile was significantly higher than other quartiles. BMI showed decreasing trend as FEV1 (%) increased, except for the 3rd quartile. The BMI of the 4th quartile was significantly lower than the other quartiles. The proportion of current smokers, sex, mean systolic BP, hsCRP, and HbA1C was not significantly different among the 1st to 4th quartiles (Table 2).

### 3.2. Comparison of Carotid Plaque, Thickened IMT, PWV (%), and Uric Acid according to the Quartiles of FEV1 (%)

We analyzed the trend for the carotid plaque, thickened IMT, PWV (%), and uric acid by the FEV1 (%) quartiles (Table 3). The percentage of carotid plaque, thickened IMT, and PWV (%) had no consistent trend and statistical significance from the 1st to 4th quartile. The serum uric acid decreased as the FEV1 increased but had no statistical significance.

### 3.3. Odds Ratio for the Risk of Carotid Plaque and Thickened IMT according to the Quartiles of FEV1 (%) after Adjustment

The odds ratio (OR) of the 4th quartile was considered as 1. In model 1, the adjusted OR of the presence of carotid plaque was lower (adjusted OR = 0.628, 95% CI 0.409–0.964, and *p* = 0.034) in the 2nd quartile than in the 4th quartile, and there was no significant relationship among other quartiles (Table 4). The OR of the thickened IMT had no consistent trend and statistical significance. In model 2, there was no significant relationship between carotid plaque or thickened IMT and FEV1 quartiles (Table 4).

### 3.4. Linear Regression Analyses of PWV (%) and Uric Acid with FEV1 (%)

**Figure 2:**
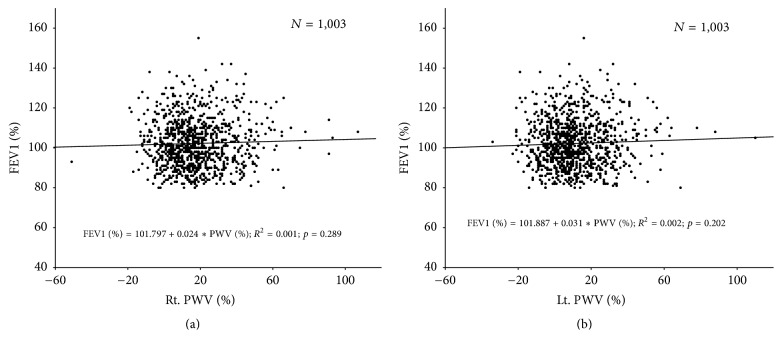
Simple linear regression analysis showing relationships of FEV1 (%) with Rt. PWV (%) (a) and Lt. PWV (%) (b).

**Figure 3:**
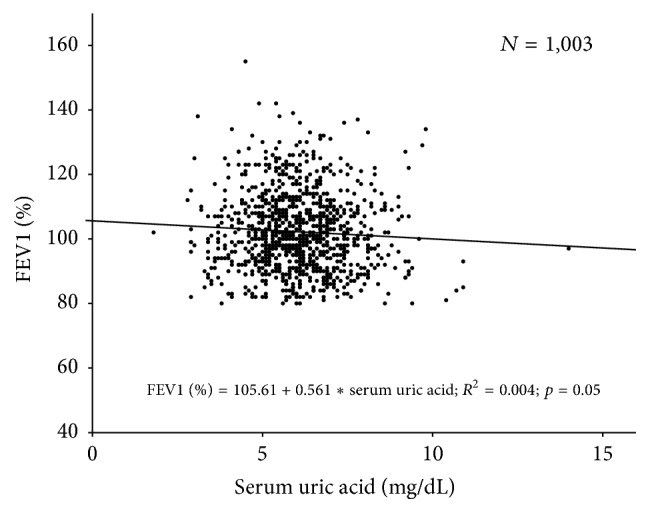
Simple linear regression analysis showing relationships of the FEV1 (%) with serum uric acid.

In Figures 2 and 3, simple linear regression analysis was used without adjusting other factors for statistical analysis. In Table 5, multiple linear regression analysis was used with adjusting multiple factors for statistical analysis.

## 4. Discussion

We hypothesized that as FEV1 decreases, CV risk would increase in general population without airflow limitation. Despite the large sample size of this study, FEV1 had no significant relationship with CV risk factors including carotid plaque, thickened IMT, PWV (%), and uric acid after adjustment of confounding factors including age, BMI, smoking status, BP, cholesterol, HbA1C, and hsCRP. The age of the 4th quartile (higher FEV1) was the highest and the BMI was the lowest (Table 2). This suggests that relatively healthy old survivors could be included, which could influence the results of this study.

The methods of measurement of PWV are variable. Carotid-femoral pulse wave velocity (cfPWV) is the most validated and is regarded as the standard technique to measure arterial stiffness. But primary use of cfPWV in clinical fields is limited because of its difficulty to be performed. Therefore, our health examination program included baPWV, which is a popular modality of quantification of arterial stiffness and which has proven to be predictive of CV events as well as mortality [26–28].

As the FEV1 increased, percentage of carotid plaque, thickened IMT, and PWV (%) had no consistent trend (Table 3). Serum uric acid decreased as the FEV1 increased; this can be explained with uric acid as a CV risk factor [16]. A recent study reported the lung protective effect of uric acid in COPD patients; a low level of uric acid was related to higher risk of COPD and lung cancer [17]. We had intended to confirm the relationship of FEV1 (%) with uric acid either as a CV risk factor or as a lung protective factor, but we could not find any relationship between uric acid and FEV1 (%) in subjects without airflow limitation. We thought that this relationship was not valid basically in subjects without airflow limitation or the relationship could be disturbed due to unknown variables.

Many studies reported the connection between airflow limitation and increased CV risk with systemic inflammation [6–8, 10]. In the progression of atherosclerosis, continued systemic inflammation could be related to recruitment and activation of macrophages and lymphocytes, which produce cytokines, chemokines, and growth factors that can induce further damage of the vessels and aggravate atherosclerosis [9]. Also, in the lung environment in COPD patients, reactive oxygen species and cytokines from alveolar macrophages, lymphocytes, and bronchial epithelial cells cannot only damage the lung structures leading to airflow limitation but also contribute to other inflammatory conditions, such as CV diseases [29, 30]. One study reported the increased hsCRP in untreated COPD patients compared to healthy controls and an inverse relationship of FEV1 (%) with the thickness of carotid IMT and serum hsCRP [10].

Based upon the previous results that reduced lung function was related to increased CV risk in COPD patients, we thought that this concept could be applicable in subjects without airflow limitation. A few previous studies reported that decreased pulmonary function was related to CV risk factors in non-COPD patients but had some limitations [31, 32].

One study reported the significant relationship between FEV1 and thickened carotid IMT and presence of carotid plaque in 1,625 subjects divided into 3 quartiles by FEV1 [31]. But there was a relationship only between the 1st quartile (lowest FEV1) and 3rd quartile (highest FEV1), and the 1st quartile included subjects with airflow limitation represented as FEV1 (pred.) <80%. So their results concerning the relationship between FEV1 and carotid IMT could have been influenced by airflow limitation.

In another small study of 27 asymptomatic ex-smokers, there was no association between FEV1 and thickened carotid IMT, but worse ventilation defect percent (VDP) measured by ^3^He magnetic resonance imaging (MRI) was related to carotid atherosclerosis measurements including IMT, vessel wall volume, and total plaque volume [32].

These results imply that ^3^He MRI can be a sensitive predictive method for detecting CV risk factors that cannot be detected by spirometry or computed tomography scan in ex-smokers without airflow limitation. This can be a novel suggestion but the interpretation has to be done with caution due to its limited sample size. However, the result that FEV1 was not related to carotid IMT but VDP on MRI related to it in the study is noteworthy.

The strength of our study is that we completely excluded subjects with airflow limitation and tried to clarify the relationship between FEV1 and CV risk factors in subjects without airflow limitation after adjustment of confounding factors. Furthermore, the sample size of this study was larger than the previous studies.

However, our study showed no relationship between FEV1 and CV risk factors. We have to consider the limitations of the study that could have influenced the results. First, this was the cross-sectional study that included only subjects that had undergone the health examination. Therefore, we reviewed the data retrospectively so we could not obtain the time sequence of the decreased lung function and increased cardiovascular risk. In addition, self-reporting of smoking habit is prone to bias; hence, cotinine measurement should be included in future studies. Second, in our study, as the FEV1 increased, the mean age of the each quartile also increased and BMI decreased. We thought this could imply that more healthy old subjects compared to general population were enrolled in this study. Third, many important confounding factors were adjusted in our study, but some other factors that could affect the results might not have been considered, such as underlying comorbidities. Fourth, we did not conduct postbronchodilator spirometry tests because of the limitation of healthcare examination program. But in most previous related studies, prebronchodilator spirometry tests have been done and we initially excluded the subject with self-reported asthma or COPD. Fifth, this study focused on a Korean population. Therefore, it is unclear whether these results are applicable to a Western population in which mechanisms and risk factors for CVD may differ. Furthermore, 96.7% of the study subjects were men, which limits applicability to the general population. Future study including more female subjects will be needed to confirm these findings.

In conclusion, the pulmonary function represented as FEV1 did not have significant association with CV risk factors, such as presence of carotid plaque, PWV abnormality, thickened carotid IMT, and uric acid after adjustment of confounding factors in a general population without airflow limitation. This implies that a relationship among them does not exist in subjects without airflow limitation or that FEV1 may be insensitive to the detection of the association with CV risk factors in general population without airflow limitation.

Studies reporting the relationship between pulmonary function and cardiovascular risk of subjects without airflow limitation have been scarce. Therefore, this study provides meaningful data. Further large-scale prospective studies are needed to clarify the relationship and for finding the cost-effective diagnostic approaches in the subclinical state.

## Figures and Tables

**Table 1:** Baseline characteristics of the participants.

Participants, *n*	1,003
Mean age, years	47.82 ± 9.27
Male	970 (96.7)
BMI, kg/m^2^	24.46 ± 3.22
Nonsmoker	272 (27.1)
Smoker	457 (45.6)
Ex-smoker	274 (27.3)
Uric acid, mg/dL	6.05 ± 1.32
hsCRP, mg/dL	0.26 ± 3.23
HbA1C, %	5.66 ± 0.98

Values are presented as means ± SDs or as numbers (%). BMI, body mass index; hsCRP, high-sensitivity C-reactive protein; HbA1C, hemoglobin A1C.

**Table 2:** Baseline characteristics by FEV1 (% pred) quartiles.

Values	FEV1 (% pred) quartile				*p* value
	1st (80–93) *n* = 251	2nd (94–100) *n* = 239	3rd (101–109) *n* = 261	4th (110–155) *n* = 252	
Age, years	46.54 ± 8.68	46.91 ± 8.31	47.32 ± 9.68	50.46 ± 9.80	<0.001
Male	243 (96.8)	232 (97.1)	254 (97.3)	241 (95.6)	0.518
BMI, kg/m^2^	24.56 ± 3.15	24.53 ± 2.86	24.85 ± 3.93	23.86 ± 2.72	0.003
Current and ex-smoker	186 (74.1)	176 (73.6)	187 (71.6)	182 (72.2)	0.542
Systolic blood pressure, mmHg	124.43 ± 15.17	121.95 ± 13.76	122.85 ± 13.85	123.93 ± 15.13	>0.05
hsCRP, mg/dL	0.21 ± 0.46	0.14 ± 0.25	0.13 ± 0.19	0.58 ± 6.42	>0.05
HbA1C, %	5.68 ± 1.09	5.72 ± 1.07	5.67 ± 1.01	5.59 ± 0.73	>0.05

Values are presented as means ± SDs or as numbers (%). BMI, body mass index; hsCRP, high-sensitivity C-reactive protein; HbA1C, hemoglobin A1C.

**Table 3:** Percentage of carotid plaque, thickened IMT, value of PWV (%), and uric acid by FEV1 (% pred) quartiles.

Values	FEV1 (% pred) quartile				*p* value
	1st (80–93) *n* = 251	2nd (94–100) *n* = 239	3rd (101–109) *n* = 261	4th (110–155) *n* = 252	
Carotid plaque	60 (23.9)	73 (30.5)	76 (29.1)	73 (29.0)	0.273
Thickened carotid IMT	20 (8.0)	19 (7.9)	23 (8.8)	29 (11.5)	0.158
Right PWV, %	17.51 ± 15.72	17.14 ± 15.33	17.54 ± 18.04	18.67 ± 17.34	>0.05
Left PWV, %	11.20 ± 14.49	9.49 ± 13.52	11.05 ± 17.77	12.07 ± 16.57	>0.05
Uric acid, mg/dL	6.21 ± 1.45	6.01 ± 1.35	6.01 ± 1.21	5.94 ± 1.22	>0.05

Values are presented as means ± SDs or as numbers (%). FEV1, forced expiratory volume in 1 second; IMT, intima-media thickness; PWV, pulse wave velocity.

**Table 4:** The risk of presence of carotid plaque and thickened carotid IMT by FEV1 (% pred) quartiles.

	FEV1 (% pred) quartile		1st (80–93) *n* = 251	2nd (94–100) *n* = 239	3rd (101–109) *n* = 261	4th (110–155) *n* = 252
Presence of carotid plaque	Model 1	Odds ratios	0.898	0.628	0.754	1
		95% CI	0.579–1.393	0.409–0.964	0.493–1.152	
		*p* value	0.630	**0.034**	0.192	
	Model 2	Odds ratios	0.961	0.655	0.791	1
		95% CI	0.612–1.507	0.422–1.018	0.512–1.221	
		*p* value	0.961	0.060	0.290	

Presence of thickened carotid IMT	Model 1	Odds ratios	0.909	0.929	0.850	1
		95% CI	0.475–1.740	0.484–1.783	0.454–1.591	
		*p* value	0.774	0.825	0.612	
	Model 2	Odds ratios	0.895	0.874	0.769	1
		95% CI	0.462–1.734	0.448–1.706	0.403–1.465	
		*p* value	0.742	0.693	0.424	

Statistical method: multiple logistic regression analysis.

Model 1: adjustment for age, BMI, and smoking status.

Model 2: adjustment for age, BMI, smoking status + LDL, HDL, SBP, HbA1C, and CRP.

**Table 5:** The relationship between FEV1 (% pred) and PWV (%) or serum uric acid level.

	Model	Adjusted *R* ^2^	*β* coefficient	*p* value
Right PWV (%)	Model 1	0.020	0.010	0.764
	Model 2	0.220	0.033	0.236
Left PWV (%)	Model 1	0.021	0.013	0.689
	Model 2	0.220	0.037	0.192
Serum uric acid level	Model 1	0.061	−0.017	0.578
	Model 2	0.087	−0.017	0.584

Statistical method: multiple linear regression analysis.

Model 1: adjustment for age, BMI, and smoking status.

Model 2: adjustment for age, BMI, smoking status + LDL, HDL, SBP, HbA1C, and CRP.
